# Supplementary material for: The Association of Tumor Immune Microenvironment of the Primary Lesion with Time to Metastasis in Patients with Renal Cell Carcinoma: A Retrospective Analysis
Source: Cancers (Basel). 2022 Oct 26;14(21):5258. doi: 10.3390/cancers14215258 (PMC9656369; doi:10.3390/cancers14215258)
Supplement: Supplementary file 1 [file cancers-14-05258-s001.zip › cancers-1968171-supplementary.pdf]

Supplemental Material

Table S1. Association between PD-L1 status, immunophenotype, and WHO/ISUP grade.

(A) PD-L1 status, immunophenotype, and WHO/ISUP grade.

**Characteristic**			**PD-L1 Negative (N = 334)**		**PD-L1 Positive (N = 234)**	
**Immunophenotype**	Desert	(N = 242)	220	90.9%	22	9.1%
	Excluded	(N = 288)	108	37.5%	180	62.5%
	Inflamed	(N = 38)	6	15.8%	32	84.2%
**WHO/ISUP grade**	Grade 1/2	(N = 215)	168	78.1%	47	21.9%
	Grade 3	(N = 215)	118	54.9%	97	45.1%
	Grade 4	(N = 138)	48	34.8%	90	65.2%

(B) Immunophenotype and WHO/ISUP grade.

**Characteristic**			**WHO/ISUP Grade**					
			**Grade 1/2 (N = 215)**		**Grade 3 (N = 215)**		**Grade 4 (N = 138)**	
**Immunophenotype**	**Desert**	**(N = 242)**	127	52.5%	80	33.1%	35	14.5%
	**Excluded**	**(N = 288)**	85	29.5%	124	43.1%	79	27.4%
	**Inflamed**	**(N = 38)**	3	7.9%	11	28.9%	24	63.2%

ISUP, International Society of Urologic Pathologists; PD-L1, programmed death ligand 1; WHO, World Health Organization.

Table S2. Comparison of baseline characteristics at the time of initial diagnosis by time to metastasis (≤ 12 months vs. > 12 months).

Characteristic, n (%)	≤ 12 mo (N = 388)	> 12 mo (N = 180)	Total (N = 568)	*p*-Value[a]	Standardized Difference
**Sex**					
Male	298 (76.8)	136 (75.6)	434 (76.4)	0.744	0.0
Female	90 (23.2)	44 (24.4)	134 (23.6)		0.0
**Age**					
Mean (standard deviation)	63.2 (11.0)	62.7 (9.8)	63.0 (10.6)	0.376	0.1
Median [range]	64.0 [23, 87]	64.0 [32, 84]	64.0 [23, 87]		
**Age category**					
< 65 y	203 (52.3)	101 (56.1)	304 (53.5)	0.122	-0.1
≥ 65 and < 75 y	121 (31.2)	61 (33.9)	182 (32.0)		-0.1
≥ 75 y	64 (16.5)	18 (10.0)	82 (14.4)		0.2
**Histology**					
Clear cell	360 (92.8)	167 (92.8)	527 (92.8)	0.998	0.0
Non–clear cell	28 (7.2)	13 (7.2)	41 (7.2)		0.0
**Sarcomatoid component**					
Absent	331 (85.3)	173 (96.1)	504 (88.7)	0.0002	-0.4
Present	57 (14.7)	7 (3.9)	64 (11.3)		0.4
**Growth pattern**					
Expansive	120 (30.9)	87 (48.3)	207 (36.4)	< 0.0001	-0.4
Infiltrative	114 (29.4)	28 (15.6)	142 (25.0)		0.3
Indeterminable	154 (39.7)	65 (36.1)	219 (38.6)		0.1
**Fuhrman grade**					
Grade 1/2	99 (25.5)	86 (47.8)	185 (32.6)	< 0.0001	-0.5
Grade 3	200 (51.5)	80 (44.4)	280 (49.3)		0.1
Grade 4	89 (22.9)	14 (7.8)	103 (18.1)		0.4
**WHO/ISUP grade**					
Grade 1/2	112 (28.9)	103 (57.2)	215 (37.9)	< 0.0001	-0.6
Grade 3	160 (41.2)	55 (30.6)	215 (37.9)		0.2
Grade 4	116 (29.9)	22 (12.2)	138 (24.3)		0.4

Characteristic, n (%)	≤ 12 mo (N = 388)	> 12 mo (N = 180)	Total (N = 568)	*p*-Value[a]	Standardized Difference
**Necrosis**					
Absent	187 (48.2)	132 (73.3)	319 (56.2)	< 0.0001	-0.5
Present	199 (51.3)	48 (26.7)	247 (43.5)		0.5
Indeterminable	2 (0.5)	0 (0.0)	2 (0.4)		0.1
**Lymphovascular invasion**					
Absent	249 (64.2)	140 (77.8)	389 (68.5)	0.0006	-0.3
Present	117 (30.2)	27 (15.0)	144 (25.4)		0.4
Indeterminable	22 (5.7)	13 (7.2)	35 (6.2)		-0.1
**TIME**					
**PD-L1 expression**					
IC0	200 (51.5)	134 (74.4)	334 (58.8)	< 0.0001	-0.5
IC1	110 (28.4)	34 (18.9)	144 (25.4)		0.2
IC2	47 (12.1)	7 (3.9)	54 (9.5)		0.3
IC3	31 (8.0)	5 (2.8)	36 (6.3)		0.2
**PD-L1 expression**					
Negative[b]	200 (51.5)	134 (74.4)	334 (58.8)	< 0.0001	-0.5
Positive[c]	188 (48.5)	46 (25.6)	234 (41.2)		0.5
**Immunophenotype**					
Desert	142 (36.6)	100 (55.6)	242 (42.6)	< 0.0001	-0.4
Excluded	215 (55.4)	73 (40.6)	288 (50.7)		0.3
Inflamed	31 (8.0)	7 (3.9)	38 (6.7)		0.2

[a] *p*-values were calculated using the chi-square test for categorical variables and Kruskal-Wallis test for continuous variables.
[b] Defined as IC0. [c] Defined as IC1/2/3.

ISUP, International Society of Urologic Pathologists; PD-L1, programmed death ligand 1; TIME, tumor immune microenvironment; WHO, World Health Organization.

Table S3. Metastatic sites of synchronous and metachronous metastatic renal cell carcinoma.

(A) Synchronous vs. metachronous.

Metastatic Site, n (%)	Synchronous[a] (N = 307)	Metachronous[b] (N = 261)	Total (N = 568)	*p*-Value[c]	Standardized Difference
Lung	212 (69.1)	145 (55.6)	357 (62.9)	0.0027	0.3
Bone	98 (31.9)	68 (26.1)	166 (29.2)	0.179	0.1
Lymph node	81 (26.4)	46 (17.6)	127 (22.4)	0.026	0.2
Liver	32 (10.4)	28 (10.7)	60 (10.6)	0.550	0.0
Adrenal glands	24 (7.8)	13 (5.0)	37 (6.5)	0.222	0.1
Pleura	16 (5.2)	11 (4.2)	27 (4.8)	0.478	0.0
Brain	13 (4.2)	8 (3.1)	21 (3.7)	0.426	0.1
Pancreas	8 (2.6)	17 (6.5)	25 (4.4)	0.042	-0.2
Kidney	8 (2.6)	9 (3.4)	17 (3.0)	0.465	0.0
Other soft tissue	7 (2.3)	11 (4.2)	18 (3.2)	0.232	-0.1
Mediastinum	4 (1.3)	2 (0.8)	6 (1.1)	0.458	0.1
Peritoneum	3 (1.0)	8 (3.1)	11 (1.9)	0.109	-0.1
Bronchus	1 (0.3)	0 (0.0)	1 (0.2)	0.363	0.1
Breast	1 (0.3)	0 (0.0)	1 (0.2)	0.363	0.1
Duodenum	1 (0.3)	1 (0.4)	2 (0.4)	0.551	0.0
Bone marrow	2 (0.7)	1 (0.4)	3 (0.5)	0.504	0.0
Skin	3 (1.0)	0 (0.0)	3 (0.5)	0.155	0.1
Other	5 (1.6)	7 (2.7)	12 (2.1)	0.378	-0.1

[a] Defined as metastasis ≤ 3 months of initial diagnosis of renal cell carcinoma.

[b] Defined as metastasis diagnosed > 3 months after initial diagnosis of renal cell carcinoma.

[c] *p*-values were calculated using the chi-square test.

(B) By time to metastasis.

**Metastatic Site, n (%)**	**≤ 3 mo (N = 307)**	**> 3–12 mo (N = 81)**	**> 12–24 mo (N = 52)**	**> 24 mo –5 y (N = 86)**	**> 5 y (N = 42)**	***p*-Value[a]**
Lung	212 (69.1)	51 (63.0)	30 (57.7)	36 (41.9)	28 (66.7)	0.0008
Bone	98 (31.9)	18 (22.2)	17 (32.7)	23 (26.7)	10 (23.8)	0.286
Lymph node	81 (26.4)	10 (12.3)	6 (11.5)	22 (25.6)	8 (19.0)	0.025
Liver	32 (10.4)	13 (16.0)	4 (7.7)	8 (9.3)	3 (7.1)	0.318
Adrenal glands	24 (7.8)	3 (3.7)	5 (9.6)	4 (4.7)	1 (2.4)	0.267
Pleura	16 (5.2)	4 (4.9)	1 (1.9)	4 (4.7)	2 (4.8)	0.571
Brain	13 (4.2)	1 (1.2)	3 (5.8)	1 (1.2)	3 (7.1)	0.215
Pancreas	8 (2.6)	5 (6.2)	2 (3.8)	4 (4.7)	6 (14.3)	0.019
Kidney	8 (2.6)	0 (0.0)	2 (3.8)	5 (5.8)	2 (4.8)	0.187
Other soft tissue	7 (2.3)	5 (6.2)	3 (5.8)	3 (3.5)	0 (0.0)	0.183
Mediastinum	4 (1.3)	1 (1.2)	0 (0.0)	0 (0.0)	1 (2.4)	0.436
Peritoneum	3 (1.0)	3 (3.7)	1 (1.9)	3 (3.5)	1 (2.4)	0.294
Bronchus	1 (0.3)	0 (0.0)	0 (0.0)	0 (0.0)	0 (0.0)	0.596
Breast	1 (0.3)	0 (0.0)	0 (0.0)	0 (0.0)	0 (0.0)	0.596
Duodenum	1 (0.3)	0 (0.0)	0 (0.0)	0 (0.0)	1 (2.4)	0.185
Bone marrow	2 (0.7)	0 (0.0)	0 (0.0)	1 (1.2)	0 (0.0)	0.504
Skin	3 (1.0)	0 (0.0)	0 (0.0)	0 (0.0)	0 (0.0)	0.417
Other	5 (1.6)	1 (1.2)	1 (1.9)	3 (3.5)	2 (4.8)	0.385

[a] *p*-values were calculated using the chi-square test.

Figure S1. Study population.

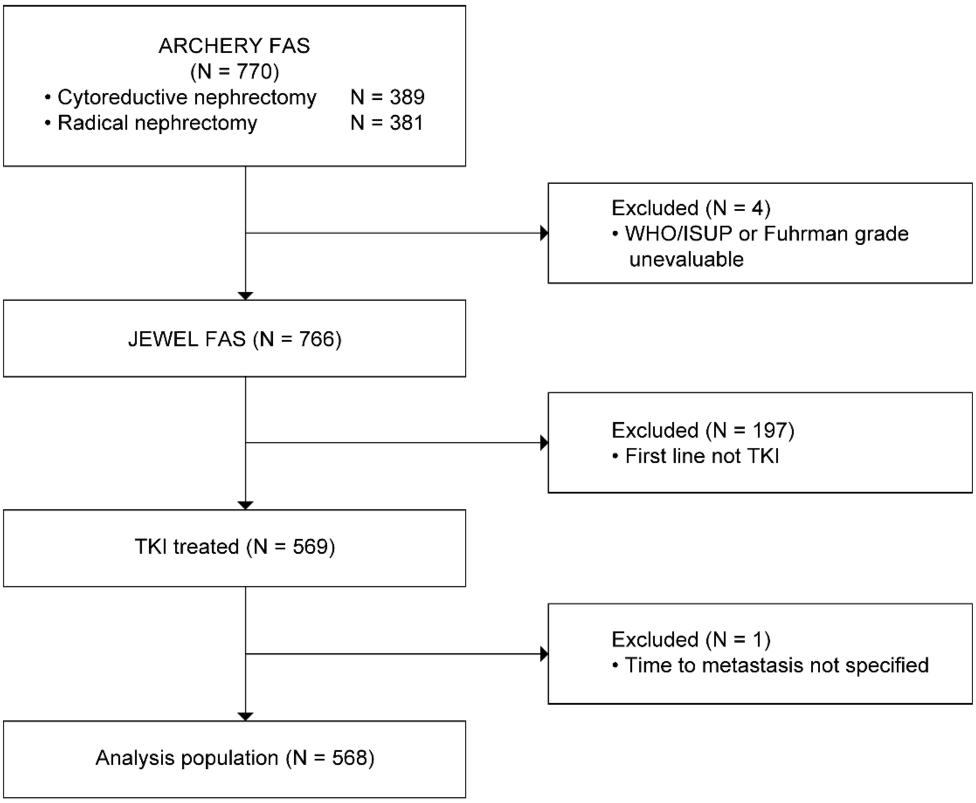

FAS, full analysis set; ISUP, International Society of Urologic Pathologists; TKI, tyrosine kinase inhibitor; WHO, World Health Organization.

Figure S2. Overall survival after first-line tyrosine kinase inhibitor treatment by time from diagnosis to metastasis (synchronous/metachronous).

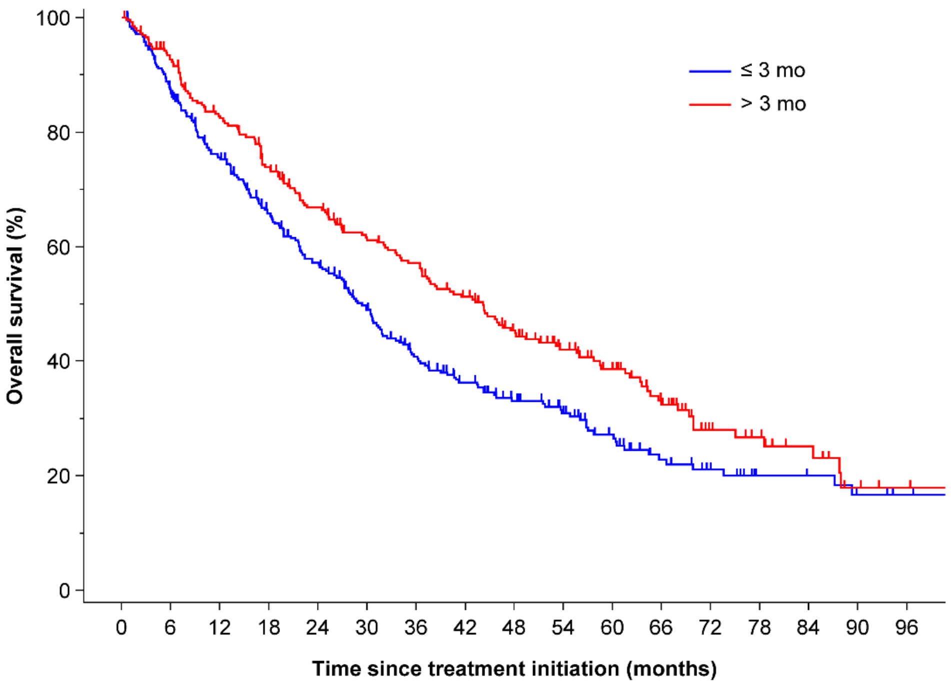

No. at risk																	
**≤ 3 mo**	307	262	220	185	158	129	102	83	68	55	41	27	21	13	12	8	6
**> 3 mo**	261	236	206	182	158	138	127	110	87	68	55	39	22	18	12	6	4

**Metastasis**	**N**	**Event**	**Median [95% CI]**	**Hazard Ratio [95% CI]**	***p*-Value[a]**
Synchronous (≤ 3 mo)	307	207	29.5 [25.1, 32.5]	Ref.	
Metachronous (> 3 mo)	261	158	44.2 [36.5, 51.1]	0.74 [0.60, 0.91]	0.0045

[a] *p*-value was calculated using Wald test for a parameter of the univariate Cox proportional hazards model.

CI, confidence interval; ref., reference.
